# Evaluation of Acromegaly patients with sleep disturbance related symptoms

**DOI:** 10.12669/pjms.37.4.4229

**Published:** 2021

**Authors:** Deniz Celik, Sezgi Sahin Duyar, Funda Aksu, Selma Firat, Bulent Ciftci

**Affiliations:** 1 Dr. Deniz Celik, Health Sciences University Atatürk Chest Diseases and Thoracic Surgery Education and Research Hospital, Department of Chest Diseases, Ankara Turkey; 2 Dr. Sezgi Sahin Duyar, Health Sciences University Ataturk Chest Diseases and Thoracic Surgery Education and Research Hospital, Sleep Disorders Center, Ankara Turkey; 3 Dr. Funda Aksu, Health Sciences University Ataturk Chest Diseases and Thoracic Surgery Education and Research Hospital, Sleep Disorders Center, Ankara Turkey; 4 Prof. Dr. Selma Firat, Health Sciences University Ataturk Chest Diseases and Thoracic Surgery Education and Research Hospital, Sleep Disorders Center, Ankara Turkey; 5 Prof. Dr. Bulent Ciftci, Yozgat Bozok University, Medical School, Department of Chest Diseases, Yozgat Turkey

**Keywords:** Acromegaly, Obstructive sleep apnea, Respiratory

## Abstract

**Background and Objectives:**

It is known that the prevalence of obstructive sleep apnea (OSA) is increased in acromegaly. Craniofacial anomalies, macroglossia, and thickening of the laryngeal wall caused by the increase in soft tissue in these patients lead to OSA. Also, the increase in growth hormone can trigger central apnea by causing a decrease in respiratory drive. Determining the polysomnographic characteristics of acromegaly patients is important to reveal the effect of these mechanisms.

**Methods:**

The demographic and polysomnographic characteristics of 33 acromegaly patients who underwent polysomnography (PSG) with suspicion of sleep disorders between 2011 and 2018 in the sleep laboratory of our hospital were retrospectively analyzed. One of the patients was excluded from the analysis because PSG was performed in the postoperative period. The remaining 32 patients with active acromegaly were grouped according to their gender and the presence of OSA and compared with statistical methods in terms of polysomnographic and clinical features.

**Results:**

OSA (AHI>5) was detected in 78.1% of 32 active acromegaly patients (18 females, 14 males) who underwent PSG with suspicion of sleep-disordered breathing. Moderate-severe OSA (62.5%) was found in most patients, and there was no difference between the sexes in terms of OSA detection rate and OSA severity. Respiratory events appear to be predominantly obstructive hypopneas. Also, the polysomnographic features of female and male acromegaly patients with OSA were found to be similar. It is seen that the OSA group is similar to the group with simple snoring in terms of body mass index (BMI), but is statistically significantly older (p=0,007). A positive correlation was found between age and AHI in pairwise correlation analysis (r:0,426 p:0,015, respectively).

**Conclusion:**

Considering that the prevalence of OSA in the population is approximately 5%, our results show that the risk of OSA in acromegaly increases, and obstructive pathways are effective in this increase. The probability of OSA occurrence and polysomnographic features between the genders are similar. Although the median BMI of the patients with and without OSA was similar, the median age was higher in the group with OSA, middle-aged acromegaly patients should be evaluated in terms of OSA even if there is no obvious obesity.

## INTRODUCTION

According to the American Association of Clinical Endocrinologists (AACE), acromegaly is defined as a disorder characterized by growth hormone (GH) hypersecretion with multisystem morbidities and increased mortality.^1^ It is also known that the prevalence of obstructive sleep apnea (OSA) increases in acromegaly. According to AACE 2011 update, the frequency of sleep apnea is approximately 70% in acromegaly with no sleep disturbances-related symptoms and more than 90% who is snoring.^1^ The craniofacial anomalies including hyperplasia of the mandible, increased length and thickness of the uvula,^2^,^3^ macroglossia, and thickening of the laryngeal wall with increased soft tissue lead to obstructive sleep apnea (OSA) in acromegaly patients.^1^,^4^ Besides, the inhibition of the respiratory center, probably due to the elevated levels of GH, may cause central sleep apnea.^5^,^6^ The pathogenesis of central apneas in acromegaly is based on the effect of GH on the respiratory center which increases sensitivity to carbon dioxide and eventually causes an arrest of the respiratory center and respiratory efforts.^6^

In this study, we aimed to reveal the polysomnographic characteristics of acromegaly patients who underwent PSG for suspicion of sleep-disordered breathing in our sleep clinic. The secondary aim was to reveal the effect of gender on polysomnographic findings in acromegaly, to compare patients with OSA with those with simple snoring in terms of age, gender, body mass index, and to determine the correlation of these factors with OSA severity.

## METHODS

The demographic and polysomnographic characteristics of 33 acromegaly patients who underwent polysomnography (PSG) with suspicion of sleep disorders between 2011 and 2018 in the sleep laboratory of our hospital were retrospectively analyzed. One of the patients was excluded from the analysis because PSG was performed in the postoperative period. The demographic and clinic characteristics of the remaining 32 patients were reviewed retrospectively. The parameters including age, gender, body mass index (BMI), symptoms, smoking status, comorbidities, and the results of diagnostic PSG were evaluated. The patients were grouped according to their gender and the presence of OSA and compared with statistical methods in terms of polysomnographic and clinical features.

### Patient selection

The diagnosis and follow-up of all cases were executed by an endocrinologist. The acromegalic patients with symptoms suggestive of sleep disorders were referred to our sleep center. After initial evaluation in the sleep clinic, the patients with snoring witnessed apnea or excessive daytime sleepiness were scheduled for PSG.

The study protocol was approved by the institutional review board of our education and research hospital (Institution Board approval date: 21.03.2019, Number: 622). All procedures performed in this study were held according to the ethical standards of the institutional review board and the 1964 Helsinki declaration and its later amendments. Only the records of patients, who had signed the informed consent for the use of their anonymous data, were analyzed.

### Measurements

All participants underwent overnight polysomnography from 11 pm to 7 am using the Compumedics Voyager Digital Imaging E-series System (Compumedics®, Melbourne, Victoria, Australia) or the Alice five system (Respironics, PA, USA). The polysomnography recordings included 4-channel electroencephalography, 2-channel electrooculography, 1-channel submental electromyography, oxygen saturation via an oximeter probe, respiratory movements via chest and abdominal belts, nasal pressure via a pressure sensor, electrocardiography, and leg movements via tibial surface electrodes. Body position was monitored by a sensor attached to a thoracic belt.

All records were manually scored according to the criteria of the American Academy of Sleep Medicine (AASM) Scoring Manual Version 2.2^7^ by a doctor who has a sleep medicine certificate from the Sleep Society in Turkey. Apnea- hypopnea index (AHI)≥5/hour was accepted as OSA. The severity of OSA was classified as follows: mild sleep apnea: 5≤AHI<15; Moderate sleep apnea: 15≤AHI<30; Severe sleep apnea: AHI≥30.

### Statistical Analyses

Data analysis was performed using the SPSS software version 15. Descriptive statistics were presented as median (25th-75th percentile). Nominal variables were presented as the number and percentage of cases. Due to the small number of patients in the study group, a non-parametric test (Mann Whitney U test) was performed to compare the distribution of the aforementioned parameters between the groups for numerical data. For determining the association between variables, the correlation coefficients, and their significance were calculated using the Spearman test. A Chi-square test was used to examine the difference between groups for categorical variables, p-value<0.05 was accepted as statistically significant.

## RESULTS

OSA (AHI>5) was detected in 25 patients (78,1%) of 32 acromegaly patients (18 females, 14 males) who underwent PSG with suspicion of sleep disorders. OSA was diagnosed in 25 patients with a median AHI of 27,4/hour (15,7-46,1/hour). Median obstructive apnea index of this group was 3 (1,08–7,2) while central apnea index was 0,15 (0-1,8). The predominant respiratory event appeared to be obstructive hypopnea in all patients. Most of the patients were presented with moderate-severe OSA (62,5%). There was no difference in OSA diagnosis rate and severity of OSA between the genders (Table-I).

**Table-I:** OSA diagnosis rate according to gender.

Diagnosis	Female (n=18. 56.3%)	Male (n=14. 43.7%)	p value
Simple snoring (AHI<5)	5 (27.8%)	2 (14.3%)	0.606
Mild OSA (AHI=5-15)	3 (16.7%)	2 (14.3%)	
Moderate-severe OSA (AHI≥15)	10 (55.5%)	10 (71.4%)	

AHI: Apnea-hypopnea index, OSA: Obstructive sleep apnea

Also, the polysomnographic features of female and male acromegaly patients with OSA were found to be statistically similar (Table-II). It was observed that the OSA group was similar to the group with simple snoring in terms of body mass index (BMI) but statistically significantly older (p=0.007) (Table-III). A positive correlation was found between age and AHI in pairwise correlation analysis (r: 0.426, p: 0.015).

**Table-II:** Clinical and polysomnographic features in patients with OSA according to gender.

	All patients (n=25) median (25.-75. percentile)	Female (n=13) median (25.-75. percentile)	Male (n=12) median (25.-75. percentile)	p-value
Age (year)	48 (36-52.5)	50 (46.5-52.5)	40.5 (30-54)	0.2
BMI (n=23)	28.7 (26.7-35.6)	30.9 (27.1-35.3)	28.2 (25.9-36.3)	0.35
TST (minute)	417 (370.1-446)	412.3 (345.6-428)	433 (397.9-468.6)	0.18
WASO (minute)	41.5 (20.9-64.8)	48 (22.4-65.4)	39 (13-61.8)	0.53
Sleep efficiency %	89.2 (81.1-93.2)	90.8 (86-92.8)	88.3 (79.9-94.4)	0.96
Sleep latency (minute)	9.5 (4.3-19)	9 (4.8-21)	10 (3.3-19.8)	0.89
REM latency (minute)	73.8 (59.1-136)	75.3 (48.8-182.5)	73.8 (67.6-130.8)	0.53
Sleep stages nREM1 %	3.1 (2.1-6)	3.1 (2.2-5.7)	3.7 (1.9-6.2)	1
nREM2 %	60.6 (50.4-67.3)	56.2 (46-69.2)	61.7 (54.9-67.1)	0.51
nREM3 %	16.8 (9.8-26.3)	23.8 (10.1-27.8)	16.6 (6.8-21.3)	0.19
REM %	16.8 (13.9-21.5)	16.3 (13.4-22.6)	17.4 (14-20)	0.96
AHI	27.4 (15.7-46.1)	24.7 (13.2-45.6)	33.9 (16.2-57.5)	0.38
nREM AHI	26.3 (9.8-53.4)	22.6 (9.7-51.6)	32 (10.1-57.5)	0.27
REM AHI	33.9 (24.9-54.8)	31.9 (17.6-43.3)	42 (25.8-58.6)	0.56
Central apnea index	0.15 (0-1.8)	0.05 (0-0.7)	0.6 (0-5.4)	0.19
Obstructive apnea index	3 (1.08-7.2)	1.7 (0.5-6)	5 (2.2-13)	0.15
Hypopnea index	16.5 (9.3-27.1)	16.5 (10-31.7	16.4 (9-24.9)	0.49
Minimum SpO_2_ %	84 (76.5-88)	84 (74.5-88)	85.5 (82.3-88)	0.76
Mean SpO_2_ %	93 (91.5-94.5)	92 (91-95.5)	93.5 (92-94)	0.97

AHI: Apnea-hypopnea index, REM: rapid eye movement, TST: Total sleep time, OSA: Obstructive sleep apnea, BMI: Body mass index, WASO: Wake after sleep onset time.

**Table-III:** Characteristics of acromegaly patients according to OSA diagnosis.

Characteristics		OSA (-) median (25.-75. percentile) (n=7)	OSA (+) median (25.-75. percentile) (n=25)	p value
Gender	Female %	27.8	72.2	0.426
	Male %	14.3	85.7
Age		34 (29-38)	48 (36-52.5)	0.007
BMI (kg/m^2)^ (n=28)		26.2 (22.9-31.8)	28.7 (26.7-35.6)	0.22

OSA: Obstructive sleep apnea, BMI: Body mass index

## DISCUSSION

Acromegaly is a disease characterized by excessive secretion of growth hormone after epiphyseal plaques have closed. The most common cause is functional pituitary adenomas. The soft tissue enlargement in these patients causes growth in the tongue and OSA as a result of thickening in the larynx wall. Studies also show that the prevalence of OSA increases in this patient group.^8^,^4^ Besides, common craniofacial anomalies in these patients contribute to the risk of OSA.^9^ The elevated levels of growth hormone may result in central sleep apnea by causing a decrease in the respiratory drive.^10^

Many irreversible comorbidities can be prevented with early diagnosis and treatment of acromegaly. Therefore, the American Association of Clinical Endocrinologists (AACE) recommended that patients who have at least two of these signs and symptoms [OSA, acral enlargement (hand, foot, and face changes), hypertension, diabetes mellitus, joint pain, fatigue, ventricular hypertrophy, systolic/diastolic dysphonia, headache, carpal tunnel syndrome, sweating, vision loss, colonic polyps, and progressive mandibular malocclusion] should be screened for acromegaly.^1^,^11^ OSA is usually diagnosed before acromegaly.^12^ Screening with insulin-like growth factor 1 (IGF-1) can provide early diagnosis and complete treatment with surgical resection which may prevent many other comorbidities. The screening for acromegaly was proven to be cost-effective, as well.^13^

Considering that the OSA prevalence in the population is approximately 5%, our results show that the risk of OSA in acromegaly is higher and obstructive pathways seem to be responsible for this increase. Therefore, patients with OSA should be screened for acromegaly in line with the AACE recommendations, and those with acromegaly should be evaluated in terms of OSA. In addition to reducing the quality of life in acromegaly, OSA may trigger the emergence of cardiovascular dysfunctions, either.^14^

Like our results, in a study by Turan et al with 30 acromegaly patients, it was found that obstructive respiratory events were more common in acromegaly patients. However, OSA was found to be more common in men.^4^ In contrast, in our study group, the prevalence of OSA was statistically similar between the genders. When the polysomnographic parameters were compared between the genders, we could not find any statistically significant difference, either. This contradictory result can be explained by the fact that the women included in this study are at an advanced age and in the postmenopausal period. A recent study showed that women with acromegaly who have OSA have a lower quality of life.^15^ Diagnosis and treatment of OSA in female acromegaly may improve quality of life, but this hypothesis should be supported by further studies.

In our study, the median BMI of the patients with and without OSA was similar, but the median age was higher in the group with OSA. Despite the lack of obvious obesity, the older patients with acromegaly seemed to have a greater risk for OSA.

In the study of Hernandez - Gordillo et al including 35 acromegalics with the mean age of 51 (39-63), the mean AHI was found to be 34 events/hour, and the mean BMI was 29 kg/m.^2^ Although they reported a slightly older group of patients with higher AHI than our cohort, they did not refer to any age-AHI-related results.^16^

Age was expressed as a predisposing factor with the onset of OSA in acromegaly.^15^ But we found no data in the literature about any correlation between age and AHI in patients with OSA and acromegaly. The moderate correlation between age and AHI may be new evidence in the literature. This evidence is important and must be tested in existing cohorts.

### Limitation of the study

Firstly, the inclusion of the symptomatic patients might have yielded a higher prevalence in our study group. As a second limitation, all-female patients were in the postmenauposal period which would minimize the gender effect on polysomnographic parameters. Nevertheless, this study contributes to determining the characteristics of female OSA in an acromegalic population and draws attention to the effect of age on OSA risk in this specific group of patients.

## CONCLUSIONS

Many complications can be prevented when acromegaly is diagnosed early and treated optimally. However, even if it is treated optimally, there is a risk of OSA in older ages. It should be taken into account that cardiovascular disorders may occur in patients with acromegaly due to the intermittent hypoxemia mechanism induced by OSA, and if OSA is detected, it should be evaluated in terms of positive airway pressure therapy. In this context, our findings support the suggestion of OSA screening for acromegaly patients, especially those who are of older ages.

### Authors Contribution:

**BC and SF** conceived, designed and editing of manuscript.

**DC, SSD, FA** did data collection & statistical analysis and manuscript writing.

**DC** takes the responsibility and is accountable for all aspects of the work in ensuring that questions related to the accuracy or integrity of any part of the work are appropriately investigated and resolved.
